# Distal Extracranial Internal Carotid Artery Stenting: A Novel Non-invasive Ultrasound Follow-Up With a Transcranial Probe

**DOI:** 10.7759/cureus.85294

**Published:** 2025-06-03

**Authors:** Giulia Frauenfelder, Salvatore Tartaglione, Gianmarco Flora, Raffaele Tortora, Gianpiero Locatelli, Alfredo Siani, Rosa Napoletano, Daniele Giuseppe Romano

**Affiliations:** 1 Neuroradiology, Azienda Ospedaliero-Universitaria San Giovanni di Dio e Ruggi d'Aragona, Salerno, ITA; 2 Stroke Unit, Azienda Ospedaliero-Universitaria San Giovanni di Dio e Ruggi d'Aragona, Salerno, ITA

**Keywords:** angiography, color doppler ultrasonography, internal carotid artery, stents, stroke, transcranial probe

## Abstract

Introduction: Distal extracranial internal carotid artery (ICA) follow-up after stenting could be challenging because of tortuosity and anatomic limitations, requiring second-line invasive exams such as CT angiography (CTA) and digital subtraction angiography (DSA). The aim of our study was to validate a new diagnostic ultrasound (US) modality, using a transcranial probe (TP), for the evaluation of the distal cervical ICA segment after endovascular stenting.

Methods: From January 2022 to February 2024, all patients stented for high extracranial carotid disease in an acute or elective setting were retrospectively enrolled. A three-month US follow-up was conducted with linear and transcranial probes. CTA and DSA were used as a standard of reference.

Results: A total of 46 patients in whom high ICA stenting was performed were included. Emergency carotid stenting was performed in 69.5%. For the evaluation of stent patency, TP demonstrated a sensitivity and specificity of 100% and 97.4%, respectively. For intrastent stenosis/decoupling in overlapping stents or pseudoaneurysm exclusion, TP demonstrated a sensitivity and specificity of 66.7% and 97.4%, respectively.

Conclusion: US evaluation using a TP with submandibular approach could be a novel, non-invasive method for stenting follow-up in distal extracranial ICA.

## Introduction

Acute ischemic stroke (AIS) represents a major problem in health, being a major cause of disability worldwide. Extracranial internal carotid artery (ICA) disease causes around 20% of ipsilateral stroke, defined as “tandem occlusion stroke.” Due to increased endovascular treatment for AIS, carotid artery stenting (CAS) for tandem lesions is being used as a necessary emergency procedure. In such a scenario, distal extracranial ICA disease requires acute treatment with CAS for about 56% of cases [1]. Procedures may include single but long stents to cover the whole plaque, and placement of overlapping stents in cases of high cervical dissection [2]. Also, pseudoaneurysms, resulting from trauma (most common), iatrogenic injury, dissection, arteriosclerosis, infection, tumor invasion, and radiotherapy, may require endovascular treatment with conventional or flow diversion stents [3-5].

Carotid color Doppler ultrasound (cD-US) is the first-line method in stenting follow-up because it is harmless, fast, relatively inexpensive, and widely available, allowing the detection of most complications (hyperplasia, restenosis, leaks, rupture).

Regarding ICA evaluation with cD-US imaging, published studies describe the standard technique as non-invasive but operator-dependent and of poor diagnostic utility for the high cervical segment due to the artery's deep course/distancing from the probe, requiring confirmation by computed tomography angiography (CTA), magnetic resonance angiography (MRA), or digital subtraction angiography (DSA) [6-8].

The aim of our study was to validate a new diagnostic cD-US, using a transcranial probe/transducer, for evaluation of flow in the distal cervical ICA segment after endovascular stenting.

This article was previously posted to the Research Square preprint on March 31, 2025 [9].

## Materials and methods

From January 2022 to February 2024, all patients treated with carotid stenting in our department were retrospectively enrolled. This research did not require IRB approval because of the retrospective and de-identified study protocol. All principles in the Declaration of Helsinki were followed, and the Italian laws on privacy (Art. 20-21, DL 196/2003), which explicitly waive the need for ethical approval for the use of anonymized use of patient data, were respected. The institution committee approved (Ref.: 12/2024) the anonymized use of patient data.

**Figure 1:**
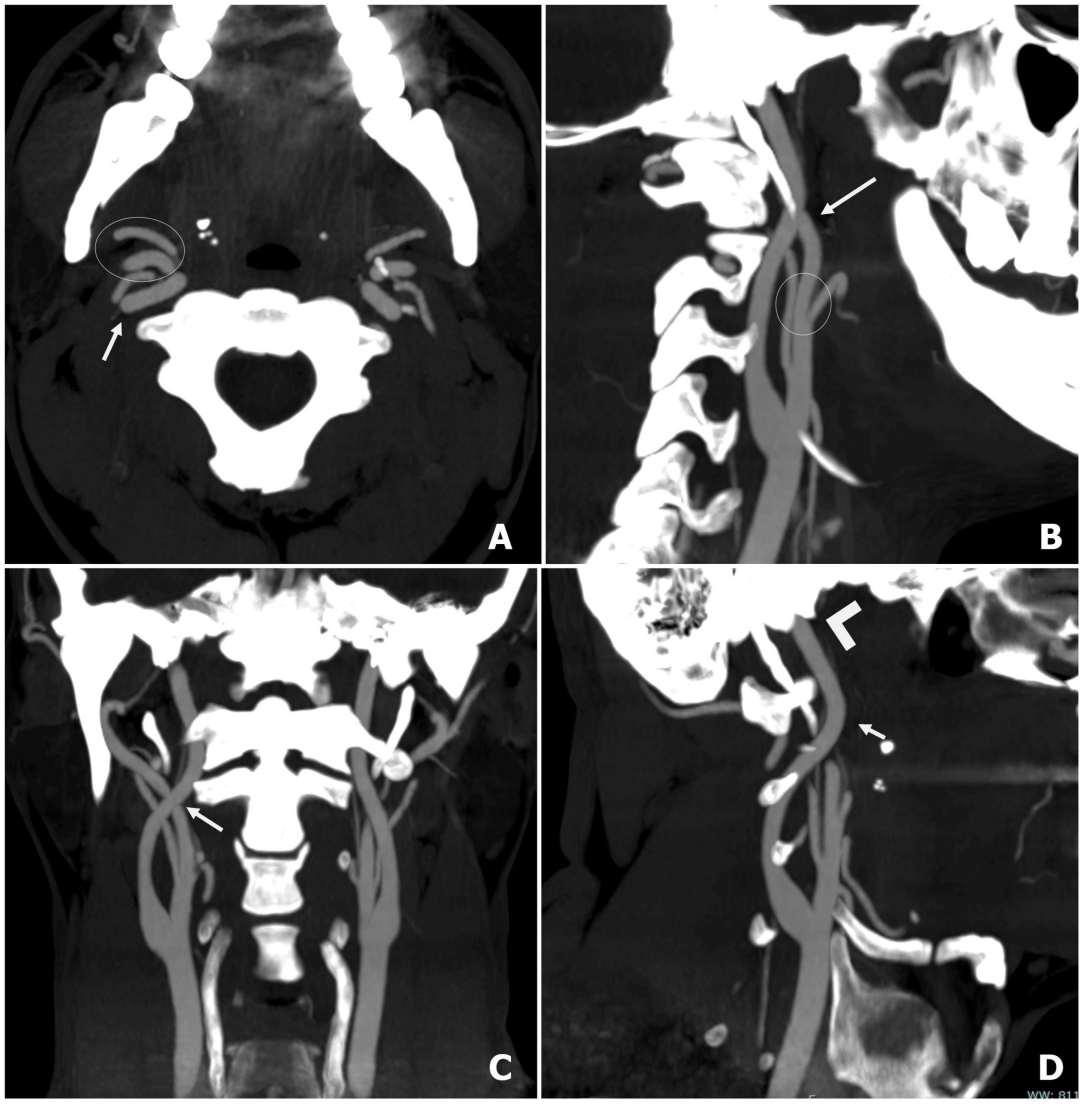
High cervical ICA segment course. Maximum intensity projection (MIP) CT angiography (CTA) shows anteromedial internal carotid artery (ICA) course (white arrow) crossing external carotid artery (ECA) branches (white circle) in axial (A), sagittal (B), coronal (C), and oblique (D) planes. ICA cervical segment ends at the level of the carotid canal (arrowhead in D).

Inclusion criteria were age ≥ 18 years, endovascular procedure with high cervical carotid stenting in emergency or elective state, and US, CTA, and/or DSA stenting follow-up at three or six months. High cervical ICA was defined as the segment that crosses behind and courses anteromedially to the main external carotid artery trunk, ascending before the carotid canal (Figure 1) [10].

**Figure 2:**
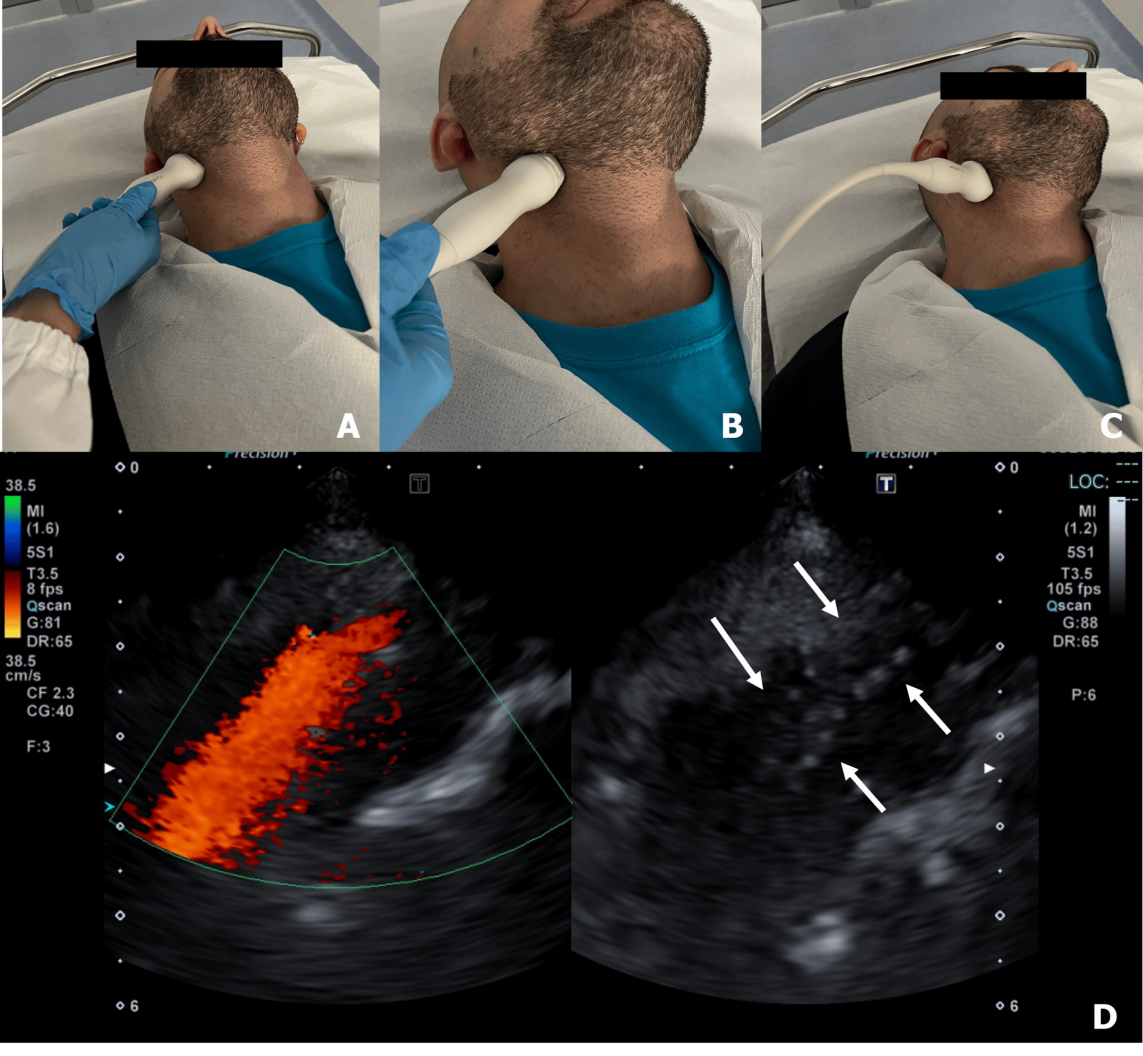
Transcranial probe position for high ICA segment evaluation. Transcranial probe position in longitudinal (A) and transverse (B) planes. Higher cervical segment (pre-petrous) could be insonated by turning the head upwards and contralaterally (C). Internal carotid artery (ICA) imaging with transcranial probe after stenting (D): ICA flow is identified in a straight vessel with distancing flow; also, stent meshes are visible (arrows).

Examinations were performed with the use of a Philips EPIQ Elite system (Philips, Amsterdam, Netherlands) and a Canon Aplio a450 (Canon Medical Systems, Otawara, Japan). Follow-up US was performed with the standard linear high-frequency transducer for carotid bulb/proximal ICA and with the transcranial transducer for high cervical ICA evaluation. With the patient in a supine position (Figure 2), the probe was positioned laterally on the neck at the interested site to study stent “morphological data” with B-mode (integrity, ruptures, neointimal proliferation, decoupling in overlapping stents) and “functional/hemodynamic data” with color Doppler (patency, aliasing, leaks) [11]. Pulsed Doppler angle was maintained at 60°. Our standard set of recorded images includes sagittal and transverse images in black and white, sagittal/transverse images in color, and sagittal with duplex imaging. Follow-up CTA was conducted after intravenous injection of 50 mL of iodinated contrast with arterial and venous scanning from the aortic arch to the brain vertex. Follow-up DSA was performed with radial or femoral access and selective contrast injections of stented ICA with dedicated projection. Both CTA and DSA were used as a standard of reference.

Transcranial transducer technique

With the patient in a supine position, the probe was placed laterally on the neck at the submandibular level. We examined high cervical ICA on axial and sagittal planes with transverse and longitudinal probe inclination, respectively (Figure 2). We identified high cervical ICA by delineation of a straight vessel near the internal jugular vein with flow proceeding upward to the skull base, and by confirming that the flow velocity pattern was identical to that of the ICA, and that branching was absent. The US set up for “carotid” evaluation is reported in Table 1.

**Table 1:** Ultrasound (US) parameter optimization with transcranial probe for extracranial carotid artery evaluation.

Parameters	Optimization
Frame rate	1
Frequency	2.3
C focus (CF)	75%
C gain (CG)	40
Dynamic range (DR)	65
Pulse repetition frequency (PRF) (scale)	20.9-38.5
Filter	3
Smoothing	2
Mechanical index (MI)	1.6

B-mode was used for anatomical evaluation of high cervical ICA and distal/end identification of the carotid stent. Color Doppler was used for the evaluation of flow direction, stent patency, and intra- and post-stent velocity (peak systolic velocity (PSV), end diastolic velocity (EDV)). A PSV > 300 cm/s for >70% instant recurrent stenosis was considered as a threshold velocity following CAS [12]. All cD-US were carried out by an interventional neuroradiologist (G.F.) in our Neuroradiology Department.

## Results

A total of 183 patients were treated with carotid stenting in emergency or elective settings from January 2022 to April 2024 in our department. We included 25.1% of cases (n = 46/183) in which high ICA stenting was performed. The mean age was 67 (range = 54-74) years, and 69.6% (n = 32/46) were male. Risk factors were hypertension (65.2%), dyslipidemia (76.1%), diabetes mellitus (34.8%), smoking (43.4%), and coronary heart disease (26.1%). Causes for carotid stenting were atherosclerotic stenosis (71.7%, n = 33/46), dissection (17.4%, n = 8/46), radiation-induced stenosis (6.5%, n = 3/46), and pseudoaneurysm (4.3%, n = 2/46). Emergency carotid stenting was performed in 69.6% (n = 32/46) of patients (Table 2).

**Table 2:** Patients characteristics.

Characteristics	n (%)
Total patients	46
Male	32 (69.6)
Female	14 (30.4)
Age	
Mean	64
Median	63
Range	54-74
Risk factors	
Hypertension	30 (65.2)
Dyslipidemia	35 (76.1)
Diabetes mellitus	16 (34.8)
Smoke	20 (43.4)
Coronary disease	12 (26.1)
Internal carotid artery pathology	
Atherosclerosis	33 (71.7)
Dissection	8 (17.4)
Radiation-induced	3 (6.5)
Pseudoaneurysm	2 (4.3)
Stenting procedure	
Emergency	32 (69.6)
Elective	14 (30.4)

A three-month imaging follow-up was conducted in 44 cases with cD-US using the linear and transcranial probes; six-month CTA imaging follow-up was conducted in 38 cases; and six-month DSA follow-up was conducted in three cases. Informed consent to participate in the study was obtained from participants (or their parents).

**Figure 3:**
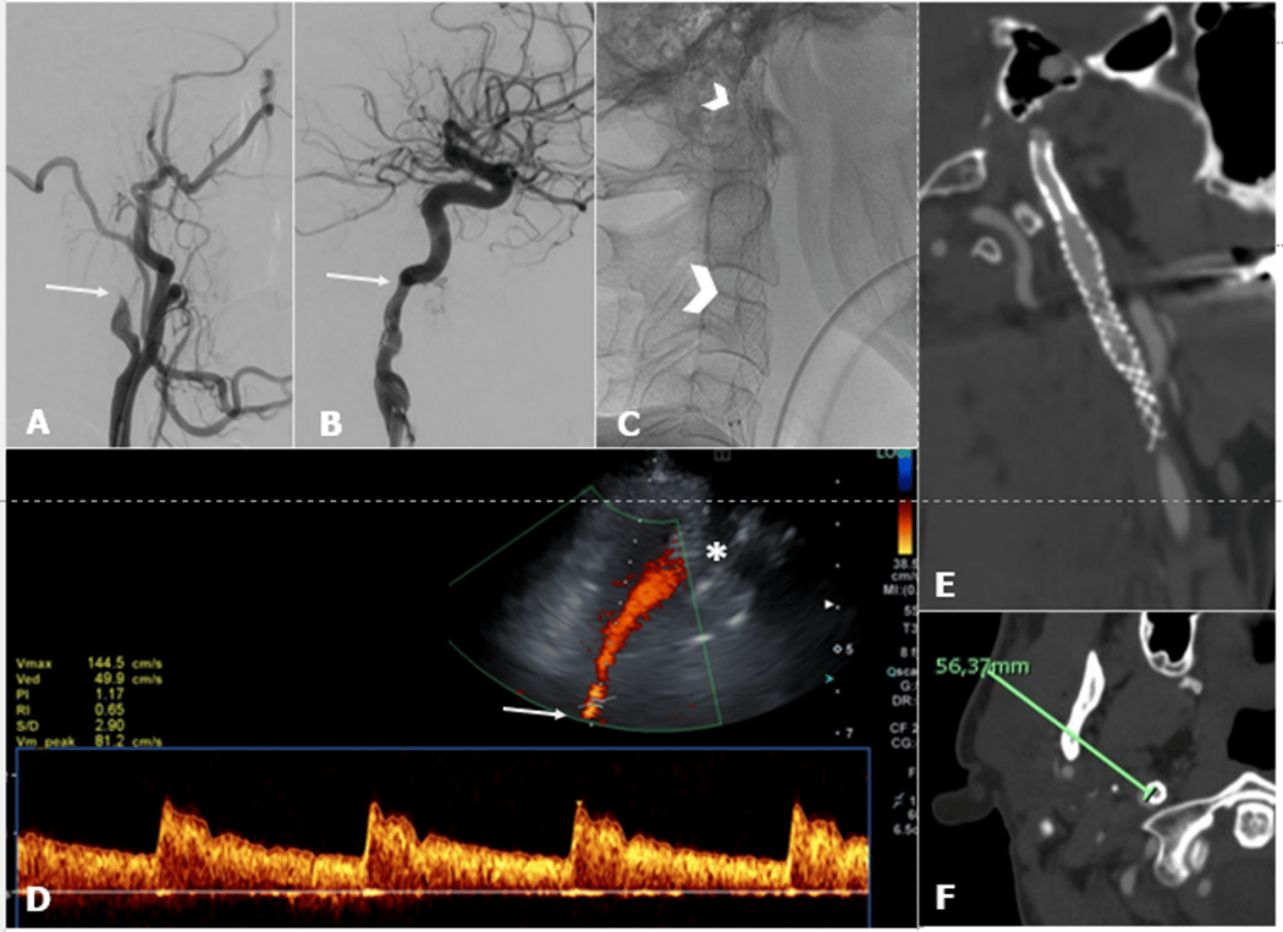
High cervical ICA stenting in tandem stroke. Right cervical internal carotid artery (ICA) dissection before (A) and after (B) mechanical thrombectomy (arrows). Acute double stenting from the carotid bifurcation to the pre-petrous ICA segment (arrowheads) (C). Color Doppler US with transcranial probe at three-month stenting follow-up (D): 6-cm depth pre-petrous ICA segment color Doppler sampling (white arrow) with velocities within limits; also, stent meshes are visible (asterisk). Six-month CT angiography (E, F) confirming stents' integrity and patency; see ICA depth in CT (F).

One patient died two days after endovascular treatment, and two patients were lost at imaging follow-up. Evaluation of morphological and hemodynamic data in the distal portion of the carotid stent was performed in 95.4% (n = 42/44) using the transcranial probe. For evaluation of stent patency (n = 41), the transcranial probe demonstrated a sensitivity and specificity of 100% and 97.4%, respectively, when compared to the standard of reference. For the evaluation of intrastent stenosis/decoupling in overlapping stents (n = 39) or pseudoaneurysm exclusion (n = 2), the transcranial probe demonstrated a sensitivity and specificity of 66.7% and 97.4%, respectively (Figure 3).

**Figure 4:**
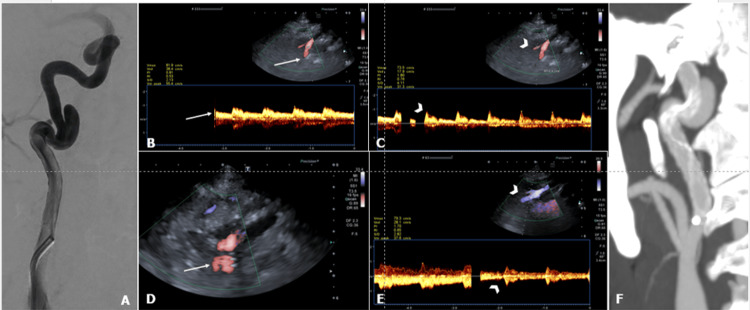
A 53-year-old man with a right high cervical ICA pseudoaneurysm. (A) Diagnostic angiography demonstrated an 8 mm wide neck pseudoaneurysm from the anteromedial high cervical internal carotid artery (ICA) wall. (B-C) Color Doppler ultrasound (US) with a transcranial probe confirmed a pseudoaneurysm with irregular arterial flow (white arrowhead in C) compared with flow from the adjacent high cervical ICA (white arrow in B). (D-E) Three-month follow-up with a transcranial probe US, demonstrating the presence of stent meshes and color signal outside the stent (arrow) along the ICA curvature; pseudoaneurysm appeared vascularized (arrowheads in E). (F) CT angiography confirmed the presence of a pseudoaneurysm with partial stent apposition on the carotid walls.

High ICA pseudoaneurysm stenting was evaluated in two cases: in one case, the presence of residual-flow pseudoaneurysm was demonstrated by transcranial probe and confirmed by CTA follow-up (Figure 4); in the other case, CTA imaging at follow-up reported a small residual neck (3 mm), which was not detected in cD-US.

## Discussion

Treatment of carotid occlusive acute disease has considerably evolved over recent years. CAS is a frequent therapeutic strategy for approaching severe carotid obstructive disease and is now widely accepted as a less invasive technique that provides an attractive alternative for many patients, particularly those with significant co-morbidities [13]. From a technical standpoint, the continuous evolution of neurovascular stent design increases the therapeutical arsenal available to deal with any carotid anatomies and different lesion characteristics (acute occlusion, dissection, pseudoaneurysm); moreover, stent flexibility technology could be helpful for treatment in high cervical ICA, where curvature must be preserved (balloon-mounted coronary stents [14], braided self-expandable stents [15], flow diversion stents [16]).

Evaluation of distal stented ICA with a linear probe could be challenging due to short neck, high carotid bifurcations, medialized/extremely deep vessel course, and the patient's poor collaboration in acute cases. Previous studies reported different approaches for the evaluation of ICA, such as convex 3.5 MHz array [17] or transoral [18], intracavitary convex array probe [19], reporting limitations due to standard convex probe dimension at submandibular space/short neck and the need for local anesthesia of the pharynx to reduce discomfort in transoral invasive approach to the pharyngeal posterolateral wall.

Our study proposed a new cD-US technique demonstrating high specificity for evaluation of patency/intrastent restenosis or residual pseudoaneurysm for the distal extracranial ICA after stenting. The use of a transcranial probe (with smaller dimensions than linear and convex probes) allows positioning in the submandibular region, reaching the pre-petrous segment of ICA with high maneuverability (particularly in short necks) and a non-invasive cervical external approach. After ICA stenting, imaging follow-up should be conducted after three and six months to verify stent patency and flow and to manage antiplatelet therapies: our protocol is based on a dual antiplatelet therapy (DAPT) for three months after treatment, while a single antiplatelet therapy (SAPT) is maintained at least for six months. So, imaging follow-up with cD-US is conducted at one, three, and six months after carotid stenting. All cD-US were performed by an interventional neuroradiologist, thus allowing a close, direct patient follow-up, through a non-invasive, fast, and low-cost method in comparison to CTA and DSA; moreover, in young subjects or patients with absolute/relative contraindications to the use of contrast medium. The aim of our study was also to raise awareness in the neurointerventional community about the importance of performing Doppler ultrasound in clinical practice, by promoting and encouraging a rapid first-line Doppler imaging, which can be helpful both in patient selection and follow-up. The limits of the study are a low number of patients and the use of US, which is defined as an “operator-dependent” modality.

## Conclusions

Standard US evaluation of distal cervical ICA flow after stenting could be challenging, mostly in high carotid bifurcations, very short necks, or extremely deep vessels. As never published before, we report high specificity of a novel, rapid, non-invasive cD-US approach with a transcranial probe for evaluation of flow in high cervical ICA after stenting. In the era of increased treatment of distal cervical ICA with stenting, our preliminary study is important because it could be helpful in patient follow-up and could contribute to reducing/avoiding more invasive and expensive examinations (CTA/DSA). Moreover, our study aims to sensitize the neurointerventional community about the importance of performing Doppler ultrasound in clinical practice.
